# Architectural Design of Biomaterial Scaffolds for Hematopoietic Stem Cell Culture

**DOI:** 10.34133/bmr.0253

**Published:** 2025-10-16

**Authors:** Naline Bellier, Yeonwoo Jang, Kevin Kent Vincent Canlas, Hansoo Park

**Affiliations:** School of Integrative Engineering, Chung-Ang University, Seoul, Republic of Korea.

## Abstract

Recently, biomaterials have been developed for ex vivo expansion of hematopoietic stem cells (HSCs). HSCs exist in highly specialized niches in the bone marrow and are extremely sensitive to their microenvironment; therefore, this review focuses on the architecture of biomaterials and its effects on HSC culture. Herein, we describe the chemical and physical components of the HSC’s niche that can be used to inform the design of biomaterials. We then summarize the effects of surface topography, structural properties, and chemical composition of biomaterials on HSC culture. Subsequently, we identify the gaps and challenges in HSC culture, informing the potential future directions that studying HSC culture on biomaterials can take.

## Introduction

Hematopoietic stem cells (HSCs) are multipotent cells with self-renewal capacity, responsible for maintaining the blood system by continuously generating all blood cell lineages [1–5]. Their clinical relevance lies in regenerative therapies such as bone marrow (BM) transplantation and gene therapy [6–8]. In BM transplants, donor or autologous HSCs are infused intravenously, where they home to the BM, engraft, and reconstitute hematopoiesis [9–12]. Additionally, HSCs and their derivatives can be modified to aid in immunotherapy with chimeric antigen receptor T cells capable of targeting specific antigens such as human epidermal growth factor receptor 2 and interleukin 13 receptor subunit alpha 2 in breast cancer and glioblastoma, respectively [13], cells capable of infiltrating immunosuppressive tumor environments and eliminating cancer cells [14]. Despite the huge clinical potential of HSCs, often in the case of autologous transplantation, the healthy population available for harvest is low and the cells are difficult to expand in vitro while maintaining stemness, which is crucial when the treatment goal requires a large number of HSCs or its derivatives; in particular, the chance of a successful BM transplant increases with number of cells in the infusion [15]. Therefore, it is crucial that an effective method of expanding HSCs while limiting their differentiation is found, and in current research, these range from the role of various growth factors/cytokines/small molecules [16–18] to biomaterials, especially biomimetic materials that create 3-dimensional (3D) microenvironments that resemble the HSC niche [19–21].

In vitro expansion of HSCs remains challenging due to their dependence on specialized BM microenvironments rich in cellular and molecular cues [22,23]. These biochemical and physical signals regulate expansion, maturation, and differentiation [24–26]. The BM contains 2 main niches: the endosteal niche, which maintains quiescent long-term HSCs (LT-HSCs), and the perivascular niche, which supports more active short-term HSCs [17,27–30]. While the perivascular niche is more relevant for expansion, understanding both is essential, especially in disease contexts [31]. CD34^+^CD38^−^ and LSK (Lin^-^Sca-1^+^c-Kit^+^) markers are commonly used to identify HSPCs in humans and mice, with additional markers distinguishing true HSCs from progenitors [17].

Current approaches to HSC culture on biomaterials come in both 2D and 3D platforms with the general aim to replicate components on the BM niche, both biochemical and physical, with more advanced platforms including organ-on-a-chip microfluidic devices [32–35]. By including multiple cellular components that are present in the niche such as osteoblasts (OBs) and endothelial cells (ECs) in distinct channels [32,33] or replicating BM structures by taking an imprint [34], organ-on-a-chip platforms that are able to closely replicate the BM microenvironment on a cellular, biochemical, or physical level have shown success in ex vivo proliferation and differentiation of HSCs. Beyond the choice in materials used for these platforms, which can already substantially affect hematopoiesis, is the architectural design of these platforms. From nanopatterning to stiffness, these architectural features have been found to affect HSCs on all levels including growth factor production and cell attachment [36,37]. Understanding the role and effect of each individual feature of the BM niche can lead to the development of highly specialized biomaterials for HSC proliferation or differentiation as well as replicating BM pathophysiology. Therefore, this review will describe the biochemical and physical properties of the BM niche before focusing on the effects of microscale and macroscale architectural features as well as surface chemistry on HSC culture. The review will then discuss current biomaterials and their applications involving HSC culture followed by future directions and challenges in biomaterial development with regard to HSC culture.

## Characteristics of the HSC Niche

The trabecular bone where hematopoietic niches exist consists of a heterogenous porous structure made up of a mineralized matrix of primarily calcium in the form of hydroxyapatite (HAp) crystals [38,39]. Blood vessels pass through these interconnected pores, which are filled with BM containing HSCs [40]. The BM niche within the trabecular bone is subdivided into sections called the endosteal and perivascular niches, characterized by their proximity to the bones inner surface and the vasculature that runs through the marrow respectively [20]. Despite being in relatively close proximity to each other and sharing HSC populations, these niches exert distinct biochemical and physical influences on HSCs that reside in them where the characteristics of the endosteal niche tend toward HSC quiescence and maintenance [41–43] while the perivascular niche encourages proliferation, differentiation, and migration in a responsive manner [44,45]. However, distinguishing the exact properties of the endosteal and perivascular niches can be complicated as there is no distinct boundary between the two; rather, the niches form a gradient with overlapping properties that includes biochemical and physical properties alike. Furthermore, while the perivascular niche provides the environment for HSC differentiation, it also contains elements that encourage HSC maintenance [46]; therefore, it is important to identify the effects of individual properties of the niches on HSCs rather than the collective effects of the environment.

### Biochemical properties

While it is agreed upon that the BM niche is a hypoxic environment, previous assumptions were on the direction of the oxygen gradient that the perivascular niche as a route of oxygen transfer would have a higher oxygen concentration than the endosteal niche. However, Spencer et al. [47] took direct measurements of the vascular and extravascular oxygen concentration in the BM niche at varying distances from the endosteum and found that the extravascular endosteal region had a higher oxygen concentration of 1.8% compared to regions >40 μm away, which had an oxygen concentration of 1.3%. Interestingly, Itkin et al. mapped the arterial blood vessels in the BM and found that the majority of arterial blood vessels resides within 40 μm of the endosteum. Furthermore, the study focused on the effect of BM vessel permeability on reactive oxygen species (ROS) in HSCs where high ROS results in lineage commitment [40].

HSCs have calcium (Ca^2+^)-related receptors in the form of G protein-coupled receptor and Ca^2+^-sensing receptor, which makes them sensitive to fluctuations in Ca^2+^ levels [48]. As an intracellular messenger, Ca^2+^ plays a role in many cellular processes including proliferation and differentiation. Inhibition of store-operated Ca^2+^ entry into HSCs results in increased expansion and progenitor commitment [49]. Due to the location of niches in the BM, it is assumed that the niche environment is rich in Ca^2+^ with a gradient between the perivascular and endosteal niches due to the process of bone resorption occurring at the endosteum. However, direct measurements by Yeh et al. of Ca^2+^ in the BM showed no substantial gradient when approaching the endosteum. Instead, higher levels of Ca^2+^ at approximately 1.0 mM were found in cavities undergoing bone resorption, with the local levels surrounding HSCs ranging from 0.7 to 2.7 mM [50]. As bone goes through constant remodeling, Ca^2+^ levels become another dynamic element in the niche that changes with mechanical loading, serum Ca^2+^ levels, and paracrine and endocrine signaling [51–54].

The extracellular matrix (ECM) is made up primarily of structural proteins collagen I, III, IV, V, and VI with the addition of glycoproteins such as fibronectin and laminin, which are also present in the BM [55–57]. Glycosaminoglycans in the form of hyaluronan, chondroitin sulfate, and heparan sulfate and proteoglycans also regulate cell behavior while maintaining tissue structure and facilitating cell–cell and cell–matrix interactions [58–60]. The majority of HSC receptors come in the form of integrins, cell surface receptors composed of α and β subunits, which mediate these cell–ECM interactions alongside cell–cell interactions [61–63]. The relationship between receptor and ECM component is not necessarily exclusive as collagen [64] and fibronectin [65] can be detected by several integrins and other cell surface receptors. These interactions between specific HSC receptors and various niche components contribute to cell adhesion, migration, and differentiation to varying degrees [65–67] and is summarized in detail by Kulkarni and Kale [68].

**Fig. 1.**
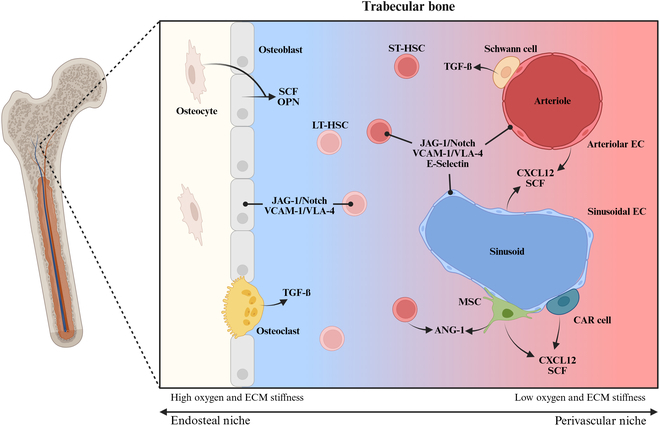
Representative diagram of cellular and molecular components of endosteal and perivascular niches within trabecular bone. Created with BioRender.

Various cytokines and growth factors that play essential roles in HSC self-renewal, proliferation, and differentiation are present in the BM niche [17]. Due to the differences in cell population responsible for secreting growth factors in the endosteal and perivascular, the factors present change depending on the niche and its population as represented in Fig. 1. The endosteal niche is made up primarily of osteogenic cells with OBs being the primary cell of interest, providing secreted factors such as stem cell factor (SCF), CXCL12 (also known as stromal cell-derived factor 1, SDF-1α), and osteopontin (OPN) [63,69–71]. Meanwhile, the perivascular niche is populated with mesenchymal stromal cells, ECs, and CXCL12-abundant reticular cells also secreting SCF and CXCL12 in addition to CXCL4 (or platelet factor 4, PF4), essential for regulating HSC function [72,73]. ECs in particular provide JAG-1/Notch interactions that are proven to be crucial to HSCs’ self-renewal capacity [74]. Other important factors that have been identified are thrombopoietin (TPO), which originates in the liver, kidneys, and BM mesenchymal stem/stromal cells (MSCs) [75,76]; transforming growth factor-β, which is secreted by nonmyelating Schwann cells [16]; and angiopoietin-1 (ANG-1) secreted by perivascular stromal cells and HSCs themselves [77].

Invariably, SCF is usually the first to be described as crucial in HSC maintenance existing both as a membrane-bound molecule on the surface of various cells and as a soluble growth factor [78,79]. Soluble SCF activates c-Kit–phosphoinositide 3-kinase/protein kinase B (PI3K/Akt) and Janus kinase/signal transducer and activator of transcription (JAK/STAT) pathways to support HSC survival and proliferation [79,80]. Meanwhile, membrane-bound SCF competes with soluble SCF for c-Kit binding, promoting HSC quiescence via vascular cell adhesion molecule-1 (VCAM-1) interactions with integrin α4β1 (VLA-4) and PI3K–actin signaling [78]. The primary of function of VCAM-1 is anchorage of HSCs to cells presenting the ligand [81], which includes OBs [82] and ECs [83]. Another important membrane-bound molecule is JAG-1 on osteogenic cells [84], ECs [74], and MSCs [85]. The presence of JAG-1 interacts with Notch-1 receptors on HSCs to promote self-renewal [86]. Interestingly, both VCAM-1/VLA-4 and JAG-1/Notch-1 interactions are present in both the endosteal and perivascular niches exemplifying a crossover of roles between niches. Further, interactions with integrins activate intracellular domains leading to downstream effects. For example, truncated OPN, found in the endosteal niche, binds with several HSC integrins that have been found to mediate different functions depending on the integrin [69]. Lack of OPN affects growth factor, cytokine, and surface expression such as vascular endothelial growth factor and Jagged-1 while also reducing HSC sensitivity to crucial cytokines [87]. Mann et al. [17] summarized in detail the numerous proteins and genes and their interactions with HSCs.

In addition to the complexities of healthy marrow, hematological malignancies result in cellular and ECM compositional changes within the BM microenvironment and are crucial to the understanding and development of biomaterial-based disease models [88]. The proliferation of malignant cells in diseases such as acute myeloid leukemia and multiple myeloma provokes BM inflammation [89,90], suppresses normal hematopoiesis [91,92], and causes immune dysregulation through altered cytokine production [93,94]. Other cell populations in the niche are also affected by the presence of malignant cells and their secretions, resulting in changes in composition of cell types and impaired signaling pathways [90,95]. This impaired signaling leads to structural changes via up-regulation of osteoclastic bone resorption and inhibition of osteoblastic bone formation [96,97] as well as vascular changes from blood vessel recruitment. The ECM can also be degraded by the release of matrix metalloproteinases by malignant cells, potentially altering the microenvironment physically and chemically [98,99].

### Physical properties

As previously mentioned, trabecular bone consists of a porous mineralized matrix with softer ECM filling in the pores resulting in a range of stiffness provided by the different matrices. Demineralized porcine BM, representative of the softer protein matrix, showed a compressive elastic modulus of 0.67 ± 0.14 MPa [100] with scanning electron microscopy imaging showing relatively uniformly oriented fibrils [101,102]. While HSCs reside in red marrow in the trabecular bone, Jansen et al. [103] identified elastic moduli of intact yellow marrow in the medullary cavity ranging from 0.25 to 24.7 kPa. Meanwhile, deproteinized BM resulted in more brittle matrices with higher compressive moduli depending on the relative density of the section being analyzed. Furthermore, untreated samples, i.e., samples containing both mineral and protein matrices, show substantially higher moduli than treated samples, implying a synergistic effect between the 2 matrices regarding the bone’s mechanical strength [104,105]. With relative density being a large factor, it is possible to individually consider the elastic moduli of the primary constituents of the mineral and protein matrices, HAp and collagen, respectively. A wide range depending on the conditions at measurement have been reported for the elastic moduli HAp and collagen at 62.75 to 176 GPa and 1 to 11.9 GPa, respectively [106–110].

**Fig. 2.**
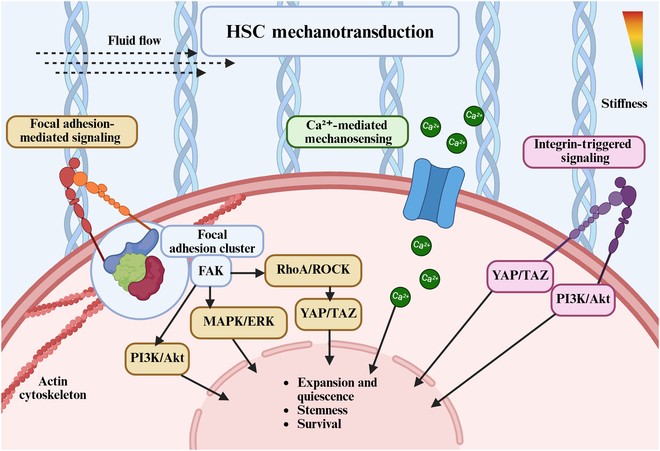
Mechanotransduction pathways regulating hematopoietic stem cell (HSC) fate. Created with BioRender.

Cells, including HSCs, express various mechanosensors that detect external biophysical cues such as matrix elasticity, fluid shear stress, and nanotopography. These sensors trigger mechanotransduction pathways, converting physical signals into biochemical responses that regulate cell behavior [111]. Integrin-based mechanosensing plays a vital role in HSC fate regulation. Upon ECM engagement, integrins cluster and recruit FAK, leading to the activation of downstream effectors including the PI3K/Akt, mitogen-activated protein kinase/extracellular signal-regulated kinase (MAPK/ERK), and Ras homolog family member A/Rho-associated coiled-coil containing protein kinase (RhoA/ROCK) pathways. These pathways converge on transcriptional regulators such as Yes-associated protein/transcriptional co-activator with PDZ-binding motif (YAP/TAZ), which translocate to the nucleus to influence gene expression. Notably, RhoA/ROCK activity also promotes cytoskeletal tension, further reinforcing YAP/TAZ activation [111,112]. Focal adhesions act as central hubs for mechanotransduction, amplifying integrin signaling and facilitating bidirectional communication between the cell and its substrate. The assembly and maturation of these complexes are sensitive to matrix stiffness, with higher stiffness generally enhancing FAK activation and downstream signaling [111]. Figure 2 provides a schematic overview of the key mechanotransduction pathways activated by stiffness, shear, and topographical inputs. These include integrin–focal adhesion–FAK cascades, ion channel-mediated Ca^2+^ influx, and their convergence on intracellular signaling modules such as PI3K/Akt and YAP/TAZ, which ultimately influence HSC fate decisions.

Importantly, changes in bone mechanics due to aging, osteoporosis, or benign bone tumors can substantially alter the marrow microenvironment. Aging, for example, can be described as a condition that is related to mechanical deterioration of the bone [113] and increased stiffness of the BM [114] while HSCs undergo cell-intrinsic decline in functionality including low regenerative potential and hematopoietic pathology [115]. Other diseases such as benign bone tumors or osteoporosis also result in physical and chemical changes within the BM [116–118]. Given the close relationship HSCs have to their microenvironment, it is likely that there is some effect on HSC function, and the microenvironment of aging, benign bone disease, and bone-weakening diseases may also play a part in HSC decline.

## Biomaterials in HSC Culture

**Fig. 3.**
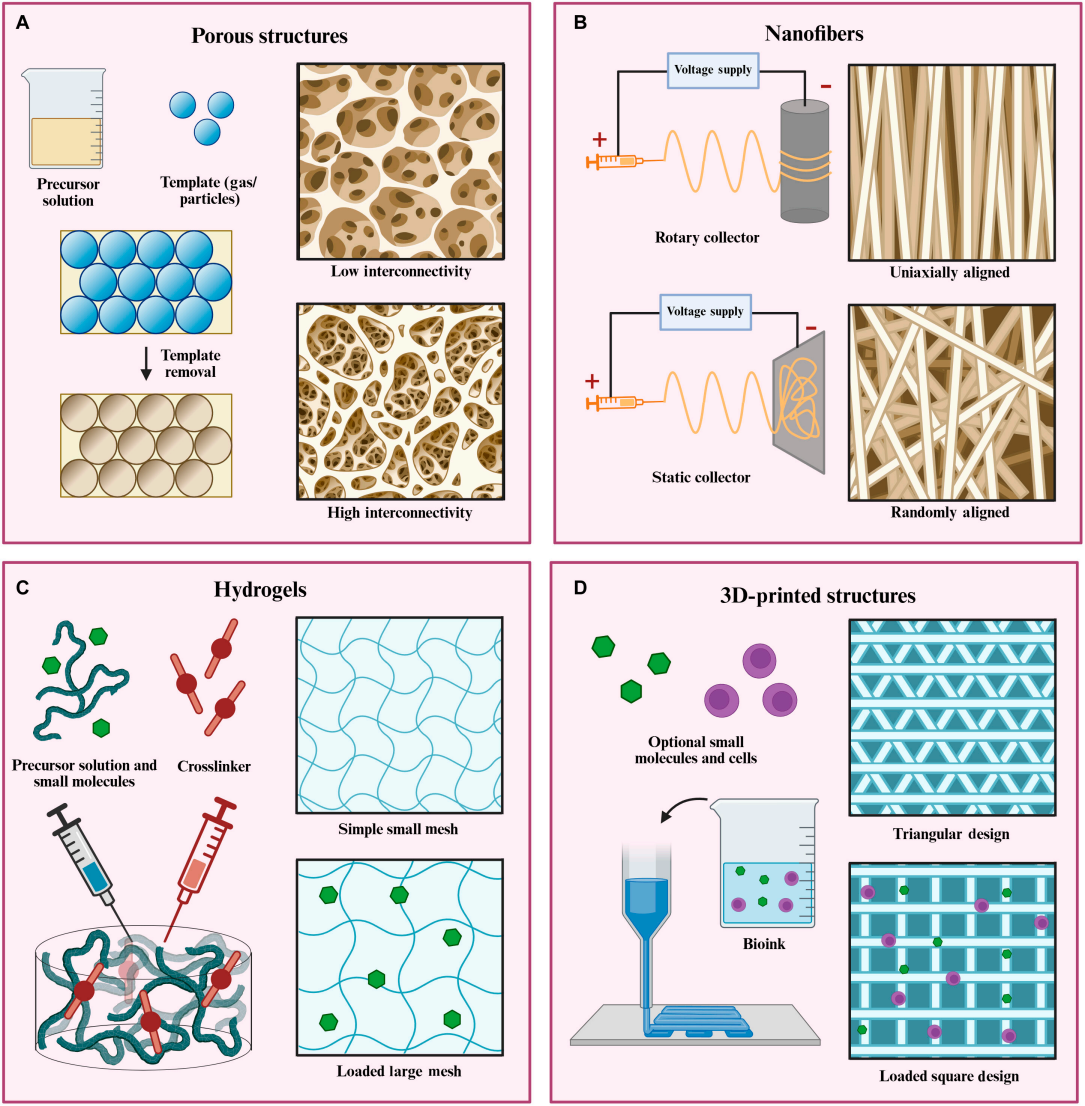
Illustrations of fabrication processes for various scaffold types including porous structures (A), nanofibers (B), hydrogels (C), and 3D printed structures (D). Created with BioRender.

Due to the complex nature of the BM microenvironment, it is popular to use decellularized and demineralized BM as a scaffold [102,119]. By doing so, researchers are able to maintain both the complexity balance of materials present and the physical structures. ECM from decellularized Wharton jelly has also been successfully used in HSC culture [120]. Beyond decellularized or demineralized tissues, scaffolds are often made from natural or synthetic polymers. Natural polymers such as collagen [121,122], alginate [123,124], and fibrin [125,126] offer biocompatibility and bioactivity but suffer from batch variability and weak mechanical properties. Synthetic polymers like poly-L-lactic acid [127,128], polyethylene glycol (PEG) [129], and polycaprolactone [125,130,131] provide greater reproducibility and stability, though they lack bioactivity and may release toxic by-products during degradation. Hybrid polymers and functionalization methods such as amination, carbodiimide crosslinking, and heparin binding combine natural–synthetic advantages and enable bioactive ligand attachment [128,132–134]. After the material has been selected, there exist several methods of scaffold fabrication that can be chosen to create a 3D microenvironment each with unique characteristics (Fig. 3).

Porous scaffolds can be created using methods such as gas foaming [135,136], lyophilization [137,138], and particulate leaching [35,139], all of which create their own characteristic pore structures with variety in pore size, homogeneity, and interconnectivity exemplified in Fig. 3. Fibrous scaffolds can be created via electrospinning or self-assembly resulting in high surface area-to-volume ratios [127,128,131]. When electrospinning, using static or rotary collectors create randomly or uniaxially aligned nanofibers. Hydrogels provide scaffolds with high water content and tunability that are created via chemical or physical crosslinking [124,129], where chemical crosslinking provides a stronger, more stable structure than physical crosslinkers, although this comes with the possibility of higher levels of toxicity. Finally, 3D printing provides a method of creating extremely high precision and resolution in scaffold geometry design using a wide range of materials both natural and synthetic, commonly using fused deposition modeling [123,140]. Other methods include stereolithography [141,142] and selective laser sintering [143,144].

In addition to these established approaches, advanced fabrication techniques such as 2-photon polymerization, multi-material 3D bioprinting, and microfluidic templating offer emerging strategies to more precisely recreate niche architecture. Two-photon polymerization enables submicrometer resolution in 3 dimensions, allowing fabrication of ECM-mimetic nanostructures such as aligned collagen fibrils or nanopores relevant to HSC mechanosensing [145]. Multi-material bioprinting expands on fused deposition modeling by enabling the spatially controlled integration of niche components such as cells, growth factors, and stiffness gradients within a single construct [146]. Similarly, microfluidic templating has been used to engineer microcavity-based scaffolds with tightly controlled dimensions and flow dynamics, which can support both autocrine signaling and dynamic perfusion [147]. Incorporating such technologies may help overcome the current trade-offs between biological complexity and reproducibility in HSC scaffold design.

Each of the 5 major biomaterial platforms used for HSC culture offers distinct structural and functional benefits. Porous sponges with interconnected pores support 3D culture, enhancing adhesion and retention; notably, polyurethane and polyHIPE scaffolds enable HSC retention while allowing egress of differentiated cells [148–150] Hydrogels, with tunable stiffness and hydration, facilitate factor delivery and encapsulation, maintaining primitive phenotypes under soft conditions [133,151]. Nanofiber scaffolds provide topographical cues that support stemness; for instance, aminated electrospun fibers enhance adhesion, and silk fibroin meshes promote expansion while preserving stemness markers [152–154]. Microcavity arrays enable control of cell–cell contact, promoting quiescence, retention, and survival [147,155]. Lastly, 3D-printed lattices offer customizable geometries and tunable mechanics, making them well-suited for niche modeling and in vivo applications [123,156].

## Effects of Biomaterial Characteristics on HSC Culture

Familiarity with the various characteristics of the BM niche allows for analysis of the roles that each have in the life cycle of HSCs. Furthermore, biomaterial architecture includes several factors such as pore size and shape, which can have an effect on the overall mechanical properties of the material as well as surface topography and roughness, all of which affect cell behavior. Surface chemistry is also essential to the design of biomaterials as it directly influences the cell’s interactions with the material. Surface modifications that can be made to the scaffold, such as conjugation of various ligands, changes in surface charge, or hydrophilicity, may have no effect on the mechanical properties of the material but ultimately affect cell behavior depending on the type of functionalization chosen and how it has been implemented. With an understanding of the characteristics of the HSC niche, the components can be individually applied and modified to both 2D and 3D cultures to direct and enhance cell behavior as necessary.

### Surface topography and roughness

Surface topography, including roughness and nanoscale patterning, impacts cell adhesion, migration, and differentiation. It is typically quantified by metrics such as average roughness and peak-to-valley height. Patterned topographies, e.g., pillars or pores, guide cell behavior through geometrical confinement. Meanwhile, surface patterning describes deliberate placement of both micro- and nanoscale structures of various designs including regular intervals of pillars, channels, and pores with more complex designs holding potential for more effective biomimicry. These features can force cells to take up certain shapes to conform to the geometry of their environment, which in turn can affect their behavior. Depending on the cell and topographical designs, surface topography can both negatively and positively influence cell adhesion, proliferation, differentiation, and migration [157–160] and even induce spontaneous differentiation [161]. Wasnik et al. [162] compared the roughness of acellular matrix (ACM) produced by BM stromal cell line, 26 ± 16 Ra, and spin-coated plates using the same ACM, 51.25 ± 3.4 Ra, and found that the spin-coated plates improved expansion of CD34^+^CD133^+^ by 44-fold on spin-coated ACM compared to 35-fold on native ACM. Beyond random nanofiber architectures, fiber alignment has gained attention as a critical topographical cue influencing HSC fate. In vivo, collagen fibrils in the BM exhibit anisotropic alignment, which plays a role in guiding cell polarity and migration [101,102]. A recent study used trench-guided electrospinning to reproducibly generate aligned nanofiber matrices with controlled orientation, offering potential for a more physiologically relevant mimic of marrow architecture [163].

While these studies highlight beneficial outcomes, the underlying mechanisms likely involve cytoskeletal and adhesion-dependent processes. In particular, Chua et al. [153,164] cultured HSCs cultured on nanofibers and observed extended filopodia, which may activate integrin-mediated signaling and cytoskeletal reorganization that supports stemness and survival. Enhanced colony-forming potential on aminated PES nanofibers may be attributed to increased contact guidance cues or nanoscale clustering of adhesion ligands [153]. Conversely, other studies report that certain topographies—such as excessively sharp or densely packed nanopillars—can disrupt membrane integrity or promote unwanted differentiation [158,161], particularly in multipotent progenitors rather than primitive HSCs [36]. These outcomes emphasize the need to tune surface architecture to avoid over-stimulation or detachment and suggest that topography alone is insufficient without biochemical support from the ECM.

Taken together, these findings confirm that surface topography can modulate HSC behavior, though the exact conditions and mechanisms remain underexplored. This is likely due to the fact that HSCs are considered weakly adherent; as such, less importance has been given to the physical characteristics of the surfaces HSCs are cultured on. However, it is clear in our later discussion of BM chemical composition that HSC adherence is highly dependent on appropriate ECM ligands and surface treatments.

### Structural properties

Porous scaffolds are commonly used in HSC research due to their similarities in structure to the HSC niches in the bone [35,165,166]. Decellularized ECM is often used to obtain a structure that duplicates BM as closely as possible [119,167]; however, due to the heterogeneous nature of bone, the porosity of the samples can vary substantially [104,105]. Porous structures have to a provide an extremely large surface area for cell attachment while allowing nutrient and oxygen transport at different efficiencies throughout the structure depending on the material and pore interconnectivity of the structure [168].

Pore geometry is often discussed in the case of 3D-printed scaffolds, which generally results in uniform cylindrical channels where internal fluid flow and wall shear stress (WSS) can be measured. Changing pore geometry affects fluid flow, which is essential in the transport of nutrient and oxygen [169–171]. It is also the primary factor in WSS values where a moderate amount can stimulate ECM production [172] or cell differentiation [173–175]; however, high WSS can lead to cell detachment [169,176]. Pore geometry may also affect cell proliferation [177] and differentiation [178] independently of fluid flow.

Pore size is known to affect cell attachment and proliferation, with cells showing better attachment efficiency in smaller pore size scaffolds [179]. However, smaller pore sizes decrease cell proliferation rates, an effect that has been attributed to contact inhibition due to the smaller surface areas available per pore [180]. Above certain pore sizes, proliferation rates also decrease as in the case of Chen et al. [181], who presented Ti6Al4V extra low interstitial structures with a range of pore sizes at 500, 600, and 700 μm, which showed decreased proliferation of BM MSCs at large pore sizes.

Relating to HSCs, Müller et al. casted 2 sizes of biofunctionalized polydimethylsiloxane (PDMS), 4-arm poly(ethylene oxide) (sPEG), and biohybrid sPEG-heparin hydrogel microcavity arrays that could fit either a single HSC cell or several ones, 15 and 40 μm in diameter, respectively. Cell proliferation was reduced in all 15-μm arrays across all materials, implicating the role of autocrine signaling in HSC quiescence [147]. Wuchter et al. created a collagen I-coated microcavity array chip with cavities 300 μm in diameter. Co-cultured alongside MSCs with a perfusion system, the HSCs showed higher expression of stem cell markers than those co-cultured on top of an MSC monolayer, indicating that the microcavity system helped them maintain stemness [155]. Notably, Marx-Blümel et al. [34] took an imprint of the BM and recreated it on a PDMS chip, achieving as close of an in vivo structure as possible and found that while it produced similar numbers of total colony forming units (CFUs) to standard 2D culture, it was more capable of maintaining multi-lineage HSCs. Overall, it is clear that pores and porous structures are beneficial to HSC proliferation and maintenance, although there is no consensus on the ideal porosity of a structure.

An important factor in biomaterial design is the stiffness or modulus of the material that can affect both the morphology and the behavior of cells, although there is reason to believe that the behavioral effects of substrate stiffness are not directly related to stiffness but rather to the morphology of the cell [182]. Cells cultured on stiffer surfaces are prone to more spreading as a result of the regulation of the cytoskeleton and the formation of focal adhesion [183] that activate mechanotransduction pathways such as YAP/TAZ and Rho/ROCK, which are responsible for cell behaviors such as proliferation, migration, and differentiation [184–187]. By creating and aorta-on-a-chip, Lundin et al. [188] blood flow-induced YAP-mediated mechanotransduction to promote HSC commitment and expansion, indicating the role of dynamic mechanical forces. While the study did not focus on substrate stiffness, Fonseca et al. [189] found that inhibition of Rho kinase in HSCs led to migration deficiency as well as alterations in cell morphology.

While HSCs have shown better ex vivo proliferation and maintenance on stiffer scaffolds when cultured alone, BM stromal cells cultured on soft gelatin methacryloyl scaffolds produced substantially more HSC niche factors, which in turn led to reversal in aging hallmarks of HSCs with which they were cocultured [114]. This study exemplifies the complex relationship that HSCs have with their niche as other studies showed that HSCs should be cultured on stiffer substrates for better maintenance of stemness. Stiffening of cellular support on which HSCs are cultured upon also increased the adhesion of cells [35].

BM-on-chip systems offer a dynamic alternative to static scaffolds, enabling long-term coculture with stromal, endothelial, and immune cells under physiological flow. These platforms replicate separate marrow niches and establish oxygen and cytokine gradients that closely mimic native niche complexity. For example, self-organizing MSC–HSPC constructs supported multilineage hematopoiesis for over 2 weeks without added scaffolds [190], while vascularized chips enabled modeling of immune cell mobilization and radiation responses [191].

Despite variability among studies, several scaffold design ranges have consistently supported HSC culture. For pore size, structures ranging from 80 to 300 μm have been associated with enhanced stemness and progenitor output [35,150,155]. Microcavity arrays around 15 μm can enforce autocrine feedback and maintain quiescence [147], whereas larger cavities (~300 μm) promote stemness in co-culture models [155]. In terms of stiffness, softer hydrogel environments of 0.2 to 1 kPa tend to support primitive HSC maintenance [192–194], while stiffer matrices between 10 and 70 kPa have shown improved adhesion, expansion, and erythropoietic potential [35,150,195]. Therefore, an optimal scaffold for primitive HSC maintenance may feature interconnected pores of 100 to 300 μm and a matrix modulus in the range of 0.2 to 1 kPa, which supports stemness under soft mechanical conditions. However, for applications requiring enhanced adhesion or lineage commitment, stiffer matrices between 10 and 70 kPa may be more suitable, particularly in co-culture systems or differentiation-driven protocols. Table 1 summarizes recent studies that focus on the effects of scaffold architecture.

**Table 1.** Effects of scaffold architecture on hematopoietic stem cell culture

Material	Structure type	Structural characteristics	Key outcomes	Ref.
SiOn/PDMS	Porous	N/A	• In vitro expansion of CD34^+^ HSCs	[34]
			• Maintained primitive phenotype	
Endotoxin-free gelatin sponge	Porous	• 30–80 μm	• Larger pores and higher stiffness enhanced HSC proliferation and stemness	[35]
		• 20–70 kPa	• Effective in vivo hematopoietic function	
Decellularized BM	Porous	• 1.6159 ± 0.2170 kPa	• Facilitated adhesion and proliferation	[102]
Decellularized cancellous bone	Porous	N/A	• Ectopic hematopoiesis	[119]
Decellularized Wharton jelly matrix	Hydrogel	N/A	• Induces proliferation and preserves stemness	[120]
			• Enhances transmigration capabilities	
Alginate /gelatin	3D printed porous structure	• Grid structure	• Increased cell proliferation; up-regulation of VLA-4/VLA-5	[123]
PLLA	Nanofiber	• Randomly aligned	• Increased proliferation, viability, clonogenicity, and CXCR4 expression	[127]
BM(PEG)_2_-PolyHIPE	Porous	• 10–130 μm	• Increased cell proliferation and egress of erythroid progenitors and neutrophils	[150]
		• 44.03 ± 24.45 kPa		
Zwitterionic hydrogel	Hydrogel	• 0.7 kPa	• Reduced ROS production	[192]
			• Promotes stemness and long-term repopulating capacity	
			• Differentiation suppression	
Gelatin	Injectable microscaffolds	• 84 μm	• Activated Notch signaling, promoting self-renewal and maintenance	[195]
		• 11.2 kPa	• Lymphoid and megakaryocytic lineage regeneration	
Collagen/PA	Porous hydrogels	• 2–35 kPa	• Rigid substrates maintain long term-HSCs	[216]
			• Softer substrates induce progenitor activity and lineage differentiation	
Ceramic	Porous structure with hollow fibers	N/A	• Creates spatial oxygen and cytokine gradients	[217]
			• Models normal and leukemic hematopoiesis	
			• Capable of continuous erythropoiesis	
CNT/HAMA	Hydrogel	• 23 ± 4 to 39 ± 6 μm	• Sustained antioxidant activity increased proliferation and preserved stemness	[193]
		• 5.0–11.4 kPa		
Alginate	Hydrogel beads	N/A	• Cyclic hydrostatic pressure enhanced HSPC migration	[218]
GelMA	Hydrogel encapsulation	• 0.2–10 kPa	• Controlled cell egress and retention	[219]
			• Autocrine/paracrine retention of cytokines	
			• Enhanced granulopoiesis	
Fibrin	Hydrogel encapsulation	• 0.78 ± 0.10 kPa and 2.72 ± 0.60 kPa	• Soft hydrogels promote HSPC expansion	[194]
Alginate, Matrigel, GelMA	Hydrogel encapsulation	N/A	• Matrigel-supported HSPC maintenance and stemness	[220]
Gel-HA/GF	Porous structure with hydrogel encapsulation	• 6.1 ± 0.1 kPa and 41.8 ± 0.3 kPa	• Supported HSPC maintenance and stemness	[166]
Gel-HA	Hydrogel encapsulation in biochip	N/A	• Directed HSPC migration and homing	[221]
• Long-term viability and phenotypic maintenance				
Silk fibrin	Nanofiber	• Randomly aligned	• Enhanced CXCR4 expression	[154]
			• Increased cell proliferation and colony-forming potential	
			• Maintenance of stemness and homing potential	

BM, bone marrow; BM(PEG)_2_, (1,8-bismaleimido-diethyleneglycol); CNT, carbon nanotubes; CXCR4, C-X-C chemokine receptor type 4; Gel, gelatin; GelMA, gelatin methacrylate; GF, graphene foam; HA, hyaluronic acid; HAMA, methacrylated hyaluronic acid; HSC, hematopoietic stem cell; HSPC, hematopoietic stem and progenitor cell; N/A, not applicable; PA, polyacrylamide; PDMS, polydimethylsiloxane; PLLA, poly-L-lactic acid; PolyHIPE, polymerized high internal phase emulsion; ROS, reactive oxygen species; SiOn, silicon oxide; VLA-4, integrin α4β1; VLA-5, integrin α5β1

### Chemical composition

The chemical composition at the surface of the scaffold can be either the same as the composition that makes up the structure or functionalized to improve its biological capabilities or influence cells as needed. Surface functionalization can be performed via covalent or noncovalent strategies that make use of chemical linkage or a natural affinity between the biomaterial and target compound [196,197]. Zwitterionic hydrogels exemplify how chemical composition can directly influence HSC behavior. Their nonfouling, redox-stabilizing surfaces suppress ROS and help preserve stemness in feeder- and serum-free conditions. Bai et al. [192] showed that such matrices support long-term repopulating HSCs by reducing intracellular ROS. A recent review highlights zwitterionic hydrogels for their excellent biocompatibility, resistance to nonspecific protein adsorption, and tunable chemistry that supports stable cell culture environments [198]. Covalent functionalization techniques such as EDC/NHS coupling, thiol–maleimide reactions, and azide–alkyne cycloaddition enable stable, site-specific immobilization of peptides and proteins. These chemistries have been used to tether SCF, SDF-1α, and TPO mimetics to hydrogels and nanofibers [199–201]. In contrast, noncovalent strategies, including electrostatic interactions, van der Waals forces, π–π stacking, and heparin-binding domains, allow reversible cytokine retention, enabling dynamic signal presentation and controlled release [134,202,203]. Covalent methods offer greater stability and control over spatial distribution and ligand density [36,200].

Different functional groups such as carboxyl, amine, and hydroxyl group can influence cell behavior and allow for further chemical modifications [35]. Notably, in the case of HSCs, aminated surfaces have been shown to dramatically increase the fold expansion of cells and total CFU and CFU-granulocyte, erythrocyte, megakaryocyte, and macrophage counts compared to carboxylated and hydroxylated surfaces. This was associated with the high amine group density inducing a positive charge that interacted with negatively charged CD34, with the interaction potentially causing downstream signaling pathways [35]. Jiang et al. [204] also noted the importance of surface charge; however, they identified that differences in chain structure could also affect HSC proliferation when modifying the surface with ethylenediamine and 2-aminoethyl methacrylate hydrochloride. Surface charge is therefore also important to consider as electrostatic interactions are crucial in the initial phase of cell attachment to a surface before integrins bond with the surface [89]. Surface charges are also capable of influencing other cell behaviors. De Jong et al. [90] found that, in addition to increasing cell proliferation, positively charged poly-L-lysine improved HSC erythropoiesis due to the increased enucleation efficiency associated with the attraction of the negatively charged nucleus to the surface coating.

ECM peptide functionalization mimics niche signals and modulates adhesion. Beyond adhesion to the ECM, other HSC receptors bind to soluble growth factors and cytokines, which can direct cell activity and distribution. Functionalization with ECM components include collagen [101,205], fibronectin [205–207], laminin [196], heparin [134,202,205], and arginylglycylaspartic acid (RGD) [36,200,201,208]. As a rule, HSCs show better adhesion and proliferation to surfaces that present ECM ligands. Interestingly, while heparin coating increases CFU numbers, desulfonated heparin was comparable to uncoated surfaces, indicating that sulfur has a role in maintaining HSC stemness [134]. Other ligands that have been conjugated to surfaces include SCF [199,200], SDF-1α [200,202], bone morphogenic protein-2 (BMP-2) [209], bone morphogenic protein-4 (BMP-4) [209], TPO mimetic peptide (RILL) [210], and Notch-activating Delta-like 1 (DLL1) [201,211] for increased surface adhesion and stemness as well as directed differentiation depending on the ligand.

A functionalized surface with a well-organized pattern of proteins can affect cell behavior and signaling [212]. Altrock et al. [36] studied the effects of integrin-binding cyclic RGD peptide distribution using gold nanopatterning where increasing ligand distance from 20 nm reduced cell adhesion. Similar methods were used by Muth et al. [164] to study the effects of fibronectin and OPN ligand distance to establish critical distances of approximately 110 and 75 nm, respectively, for cell adhesion with no effects on HSC proliferation or differentiation based on ligand type being found. Winkler et al. and Kratzer et al. [201,213] applied the concept further by applying these methods to DLL1 ligands on the surface of RGD-containing PEG-based hydrogels to enumerate ligand density necessary for HSC proliferation and induce T cell commitment, respectively. It is important to consider immobilization of factors as there can be a marked effect depending on whether the protein is soluble or immobilized. Mahadik et al. [199] found that immobilized SCF was more effective in maintaining primitive HSCs while continuous SCF promoted proliferation and differentiation. When differentiating induced pluripotent stem cell spheroids into HSCs, Bello et al. [214] found more success when BMP-4 and SCF were conjugated to GMPs included within the spheroids in achieving primitive HSCs. Lee et al. [210] chose to compare soluble and immobilized RILL where the conjugated surface resulted in comparable CD34^+^ HSC growth to TPO supplemented media. Recent studies of HSC culture with various biomaterials and biochemical modifications are listed in Table 2.

**Table 2.** Effects of biochemical modification of scaffolds on hematopoietic stem cell culture

Scaffold type	Bioactive factor/ligand	Delivery strategy	Effect on HSCs	Ref.
3D PDMS	SiOn	Surface coating	• Enhanced initial attachment and proliferation	[34]
			• Increased differentiation	
Nanopatterned hydrogel	IDSP motif and SDF-1α	Covalent nanopatterning and heparin-mediated cytokine loading	• Increased cell polarization and maintenance of stemness	[222]
Alginate hydrogel	ANG-1	Covalent conjugation	• Maintained LT-HSC quiescence • Enabled Tie-2/Ang-1 signaling without feeder cells	[124]
PolyHIPE	BM(PEG)_2_	Covalent conjugation	• Increased cell proliferation and egress of erythroid progenitors and neutrophils	[150]
PLLA nanofibrous scaffold	Selectin	Covalent conjugation	• Increased proliferation, CXCR4-mediated homing, and colony-forming potential	[223]
PCL microfiber	Collagen	Covalent conjugation	• MSC-mediated OPN secretion	[224]
			• Maintenance of HSC proliferation and stemness	
HAMA hydrogel	CNT	Encapsulated	• Sustained antioxidant activity increased proliferation and preserved stemness	[193]

ANG-1, angiopoietin-1; BM(PEG)_2_, (1,8-bismaleimido-diethyleneglycol); CNT, carbon nanotube; CXCR4, C-X-C chemokine receptor type 4; HAMA, methacrylated hyaluronic acid; HSC, hematopoietic stem cell; MSC, mesenchymal stem/stromal cell; OPN, osteopontin; PCL, polycaprolactone; PDMS, polydimethylsiloxane; PLLA, poly-L-lactic acid; PolyHIPE, polymerized high internal phase emulsion; SDF-1α, stromal cell-derived factor 1; SiOn, silicon oxide; Tie-2, TEK tyrokinase protein receptor

## Future Prospects and Challenges

**Fig. 4.**
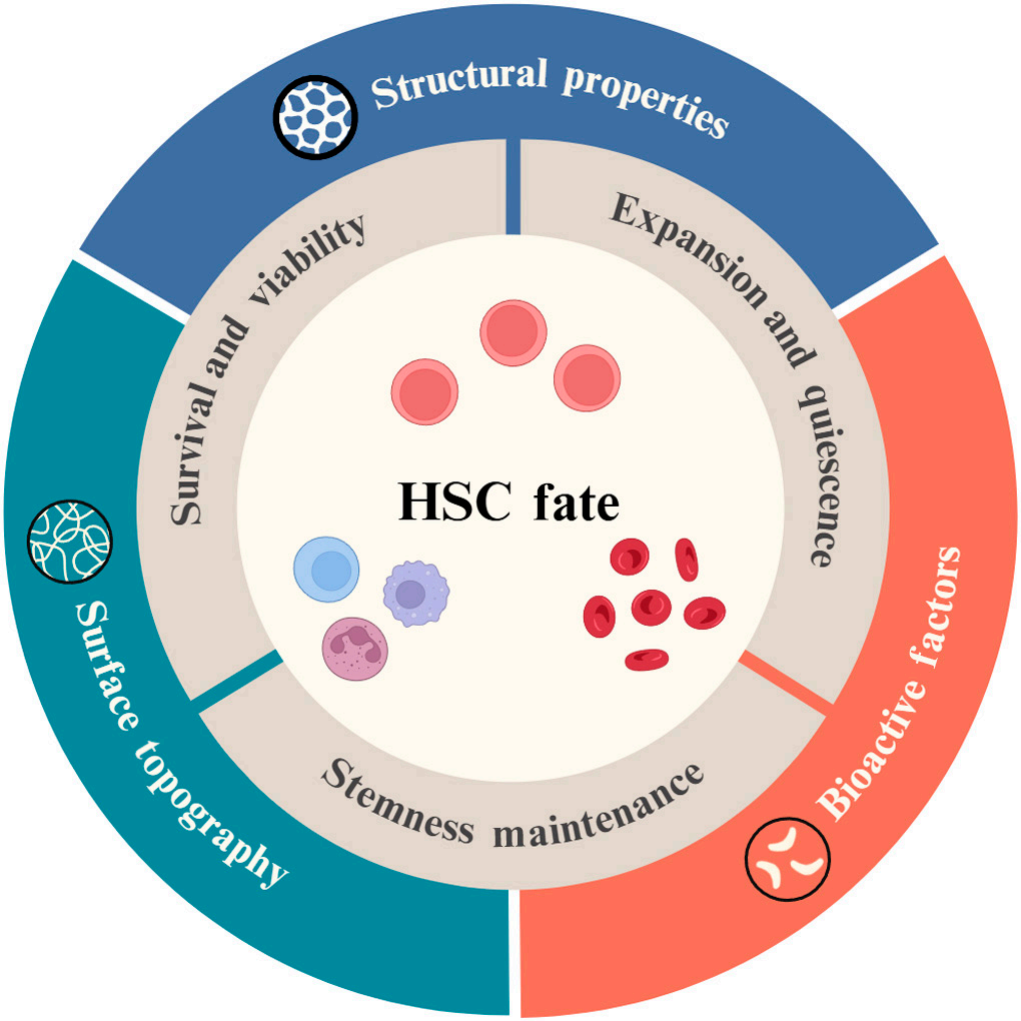
Schematic overview of how biomaterial scaffold features influence hematopoietic stem cell (HSC) fate. Created with BioRender.

Several clinical trials of BM transplant using ex vivo expansion of HSCs have had led to successful patient recoveries [215]. As a whole, exploring effects of various characteristics of biomaterials and mimicry of the HSC niche continues to be a subject of interest. However, it is clear that the study of biomaterials still has a lot to offer to the ex vivo culture of HSCs. Figure 4 provides a conceptual summary of how each of the key scaffold properties, including surface topography, structural features, and biochemical functionalization, exerts a dominant influence over distinct aspects of HSC fate such as survival, stemness, and lineage commitment. Recent studies have mostly leaned toward porous structures and hydrogels where it has been identified that smaller pore sizes limit proliferation while improving stemness; however, no consensus has been made on the ideal pore size. Furthermore, the effect of pore geometry has yet to be studied in depth. Although HSCs generally maintain their shape rather than conforming to the scaffold like other cell types, other factors such as fluid flow and nutrient diffusion may be affected and ultimately influence cell behavior. Nanofibers are underexplored in HSC research, partly because they lack the full 3D structure of porous scaffolds or hydrogels. Although random fiber alignment has shown some benefit, collagen fibrils in BM are uniaxially aligned, highlighting the potential of aligned nanofibers to influence HSC behavior.

Decellularized ECM, natural polymers, and synthetic polymers have all been used in HSC culture as well as surface coating of synthetic polymers with natural polymers; however, hybrid polymers have yet to be researched with regard to HSCs despite their abilities to make use of the advantages of both polymer types while mitigating the disadvantages. Considering the large number of both soluble factors and ECM components present in the HSC niches, the use of surface functionalization has not been taken to its limit particularly in modular functionalization approaches that enable localized presentation of multiple ligands. Methods like photopatterning, microcontact printing, or layer-by-layer assembly allow spatial control of bioactive molecules, which can recreate ECM heterogeneity or establish instructive gradients affecting HSC migration, adhesion, or lineage commitment. It has been observed that SCF, BMP-4, and RILL supplied in a continuous and conjugated manner have different effects on HSCs; however, the effects of other growth factors such as BMP-2 with a conjugated counterpart have yet to be studied despite existing in both soluble and anchored forms in the BM. Hyaluronic acid also stands out as a substantial component of the ECM that is understudied with regard to HSCs as a scaffold or a surface coating.

Beyond HSC proliferation and maintaining stemness, HSCs and BM models have many more potential applications that are as of yet unstudied. The number of lineages that HSCs can differentiate into speaks to a number of possible applications including the production of various immune cells, red blood cells, or platelets in a continuous manner. Furthermore, scaffolds as BM disease models have not been fully explored despite the changes in BM tissue and bone density in the presence of hematological disorders and more benign diseases. HSCs have been proven to be heavily influenced by their microenvironment; however, given the extreme complexity of the BM, it would be challenging to accurately emulate both healthy and diseased environments.

The use of biomaterials in the production of HSC-derived cells also suffers from the issue of scalability and long-term stability depending on the materials and functionalization. As scaffolds are scaled up in size, nutrient and oxygen supply throughout the scaffold must be taken into consideration as pores with lower interconnectivity may suffer in this regard, inadvertently depriving cells deeper within the scaffold. Precisely designed 3D-printed scaffolds with fluid dynamics simulations offer a solution in such cases. Other considerations include cost as collagen is popular for use in BM scaffolds due to its abundance in the ECM; it is more expensive than its derivative, gelatin, with other ECM materials such as fibronectin and laminin being even costlier. Furthermore, any scaffolds that use growth factors would also be extremely costly and difficult to produce for commercial use due to their instability and short life, limiting the shelf life of any BM scaffolds. Degradation of the material is also a concern in long-term and continuous cultures as the initial characteristics of the scaffold should be maintained while also ensuring that no toxic by-products occur with degradation.

To move from experimental systems toward clinical translation, it is important to align biomaterial design with practical considerations such as manufacturing scalability, regulatory approval, and compatibility with good manufacturing practice conditions. For example, while growth factor-functionalized scaffolds show promise in vitro, their clinical applicability is hindered by cost, stability, and reproducibility concerns. Translational research should therefore focus on identifying minimal essential cues or stable mimetics that can be standardized. Similarly, scaffold fabrication techniques such as 3D bioprinting—currently underutilized in HSC research—offer potential for reproducible, scalable designs when paired with computational flow modeling to optimize nutrient and oxygen delivery. The use of synthetic–natural hybrid polymers may further support clinical translation by enabling tunable degradation profiles and reducing reliance on xenogeneic components. The integration of such translational goals during scaffold design could accelerate the clinical utility of ex vivo HSC culture systems and related applications such as immunotherapy production or disease modeling. Ultimately, it is crucial to carefully consider every aspect of the scaffolds and surfaces HSCs are cultured on.

## Conclusion

In summary, HSCs reside within the trabecular bone in highly specialized and heterogeneous niches called the endosteal and perivascular niches. HSCs are sensitive to both the physical and chemical characteristics of their microenvironment, affected by the ECM stiffness, Ca^2+^ content, oxygen tension, and interactions with various other BM cells and their secretions. The heterogeneity of the environment and the sensitivity of HSCs to the said environment offer a unique challenge in recreating and studying the niche’s components and their effects on the cells. Therefore, biomaterials offer a method of studying the various niche components individually and in various combinations.

In this review, we summarized the recent studies that have taken place and discussed older studies that have paved the way for our understanding of how HSCs interact with their environment. HSC monoculture increases proliferation and maintains stemness on stiffer substrates, while the combination of softer substrates and coculture with BM-MSCs can substantially increase HSC functionality in ex vivo culture. Increased roughness and porous structures offer environments closer to that of the HSC niche that can be enhanced by the presence of ECM components found in the BM niche with integrin-binding peptides and proteins being particularly popular choices. Surface conjugated growth factors and peptides such as SCF, BMP-2, and DLL1 can be used to aid HSC maintenance or differentiation. Despite the progress that has been made in ex vivo HSC culture, biomaterials still have a role in the study of HSCs that will allow for designs of scaffolds and surface functionalization for extremely targeted purposes such as creating specific disease models or continuous production of various cell types.
